# Seasonal Variation in Leaf Extract NH_3_ Emission Potential in Fertilized Winter Oilseed Rape

**DOI:** 10.3390/plants15213411

**Published:** 2026-11-03

**Authors:** László Horváth, Tünde Alapi, Tímea Süli-Zakar, Zoltán Bozóki, Anna Szabó

**Affiliations:** 1 HUN-REN–SZTE Research Group for Photoacoustic Monitoring of Environmental Processes, Department of Optics and Quantum Electronics, University of Szeged, H-6720 Szeged, Hungary; zbozoki@physx.u-szeged.hu (Z.B.);; 2 Department of Molecular and Analytical Chemistry, University of Szeged, H-6720 Szeged, Hungary; 3 Institute of Plant Sciences and Environmental Protection, University of Szeged, H-6800 Hódmezővásárhely, Hungary

**Keywords:** *Brassica napus*, ammonia exchange, ammonium, leaf area index, canopy development, nitrogen fertilization, emission potential, seasonal dynamics

## Abstract

Seasonal changes in leaf chemistry may help characterize ammonia exchange potential in fertilized crops. We investigated leaf-extract ammonium, pH, and emission potential (Γ_extract_ = [NH_4_^+^]/[H^+^]) alongside leaf area index (LAI) in winter oilseed rape (*Brassica napus* L.) near Kartal, Hungary, in 2026. Twenty-eight pooled extracts were analyzed between February and May. Two applications of liquid urea–ammonium nitrate fertilizer each supplied approximately 70 kg N ha^−1^. Unsmoothed Γ_extract_ reached 43,087 after the February application and a smaller local maximum of 17,364 after the March application. Corresponding five-point moving-average maxima were 24,409 and 8533. LAI near the applications increased from 1.10 to 2.14. Semi-quantitative NH_3_ flux estimates combined measured concentration gradients with a mean diurnal turbulent diffusion coefficient profile obtained at the same site in 2025. Net emission increased following both applications, with estimated cumulative net emission of 13.6 kg N ha^−1^ between 18 February and 29 June. These estimates represent combined soil–vegetation exchange. Extract apoplastic purity was not independently validated, and the absence of an unfertilized control and replicated treatments precludes causal attribution. The observations motivate testing fertilizer timing relative to canopy development but do not demonstrate management efficacy.

## 1. Introduction

Industrial nitrogen fixation through the Haber–Bosch process has enabled the widespread use of mineral fertilizers and supported major increases in agricultural production [1]. The resulting expansion of reactive nitrogen inputs has also altered the global nitrogen cycle [2]. Nitrogen that is not incorporated into harvested products may remain in soil organic matter, be redistributed within agricultural systems, or be lost through leaching and gaseous pathways.

A single reactive nitrogen atom can contribute to several environmental effects as it moves between chemical forms and compartments, a sequence described as the nitrogen cascade [3]. Ammonia is relevant both as a pathway of agricultural nitrogen redistribution and as a precursor of ammonium-containing atmospheric particles. Nitrogen deposition can additionally contribute to ecosystem eutrophication and acidification [4]. Understanding agricultural ammonia exchange therefore requires attention to the chemical conditions that promote release, the pathways that permit transport, and the subsequent fate of emitted nitrogen.

Fertilized vegetation participates in ammonia exchange as a potential source and sink. Ammonia generated at the soil surface may escape to the atmosphere or interact with the canopy, while leaves can exchange ammonia through their stomata. The stomatal compensation point is the gas-phase concentration in equilibrium with the substomatal liquid phase; the direction of the stomatal exchange component depends on its relationship to the adjacent atmospheric concentration [5,6]. Soil, stomatal, and non-stomatal pathways contribute differently to the canopy-scale balance. Consequently, leaf chemistry alone does not establish either net canopy emission or total fertilizer nitrogen loss.

The chemical emission potential, Γ, is conventionally expressed as the molar ratio of ammonium to hydrogen ion concentration. For apoplastic fluid, this ratio is denoted Γ_s_ and contributes to the stomatal compensation point through the temperature-dependent ammonium dissociation and ammonia partitioning equilibria [5,7]. Both ammonium concentration and pH are therefore needed. In oilseed rape, controlled experiments have linked apoplastic chemistry to ammonia exchange and demonstrated that temperature and leaf conductance modify the response [8,9]. The ratio is a chemical indicator rather than a flux measurement.

Foliar ammonium pools are regulated by nitrogen uptake, transport, assimilation, and internal metabolism. Work on grass species has demonstrated rapid changes in leaf nitrogen pools and ammonia exchange after changes in root nitrogen supply [10]. Crop-scale assessments likewise emphasize the interaction between ammonium production and assimilation [11]. This provides a physiological basis for expecting seasonal variation, but it does not identify the mechanism responsible for a particular field observation without supporting measurements.

Existing compilations illustrate the wide range of reported stomatal emission potentials. Zhang et al. [6] compiled values across vegetation types, including large transient values after fertilization. Massad et al. [7] proposed parameterizations with a post-fertilization peak followed by relaxation toward a background level. Temporal variability has also been investigated in intensively managed grassland [12]. The literature therefore supports a dynamic treatment of emission potential, while differences in species, nitrogen supply, environmental conditions, and measurement techniques limit direct numerical comparisons.

Field observations across crop development can help assess whether repeated applications of similar nitrogen doses produce comparable chemical responses. Leaf area index (LAI) measurements provide an independent indication of canopy development against which leaf-extract ammonium and emission potential can be interpreted. However, the relevance of the extracted fluid to stomatal exchange depends on the specificity of the recovery procedure. Established extraction protocols address intracellular contamination [9,13], and pH measurements in small biological samples present additional analytical challenges [14,15].

The objective of this study was to describe the joint seasonal variation in leaf-extract ammonia emission potential, ammonium concentration, and LAI in winter oilseed rape, focusing on two equal spring nitrogen applications. We examine whether the contrasting chemical responses coincide with different canopy sizes and discuss the implications for nitrogen management. The measured ratio is designated Γ_extract_ to distinguish it from a validated in vivo stomatal emission potential. We additionally assess the seasonal timing and approximate magnitude of net field-scale NH_3_ exchange using semi-quantitative flux estimates and examine their correspondence with the leaf-extract record. These estimates provide exchange context but cannot separate soil and vegetation contributions.

## 2. Results

### 2.1. Seasonal Variation in Leaf Chemistry and Canopy Development

**Figure 1:**
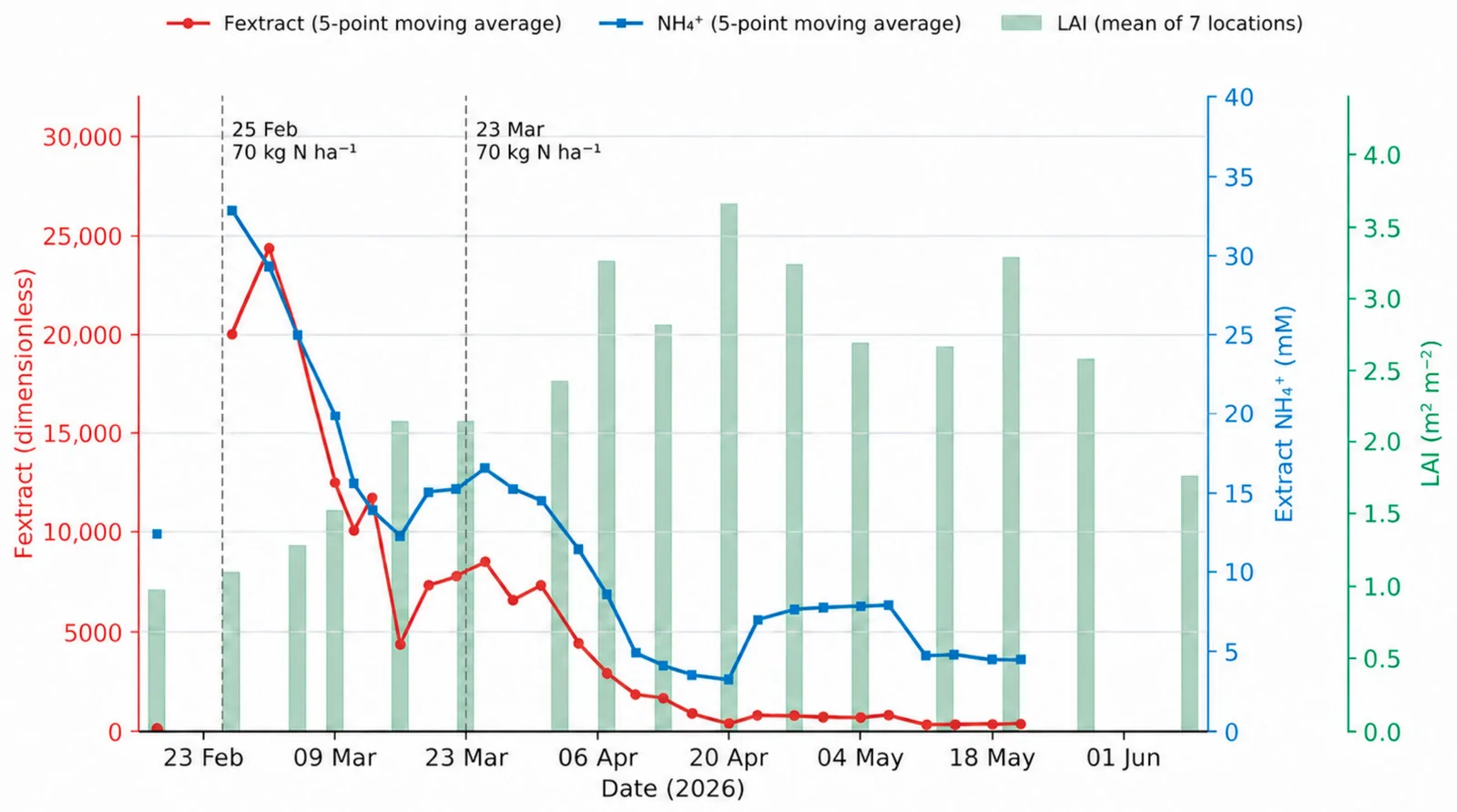
Seasonal variation in leaf-extract ammonia emission potential (Γ_extract_), ammonium concentration, and leaf area index (LAI) of winter oilseed rape. Black crosses represent the original, unsmoothed Γ_extract_ values from 28 pooled extracts, each corresponding to one sampling date. Red circles and blue squares connected by lines show the five-point moving averages of Γ_extract_ and ammonium concentration, respectively. Green bars indicate mean LAI across seven field locations on 16 measurement dates. Dashed vertical lines mark the two nitrogen applications, each supplying 70 kg N ha^−1^.

In the five-point moving-average series, Γ_extract_ was approximately 20,073 on 26 February and reached its seasonal maximum of 24,409 on 2 March, after the first urea-ammonium nitrate (UAN) application on 25 February (Figure 1). Following the second application on 23 March, a smaller local maximum of 8533 occurred on 25 March, approximately 35% of the early-season maximum. In the unsmoothed series, Γ_extract_ reached 43,087 on 26 February and a smaller local maximum of 17,364 on 25 March. These values were calculated directly from the ammonium concentration and pH of the corresponding pooled extracts. Because sampling intervals were irregular, the centered five-point moving average spans unequal time periods and may combine observations from before and after fertilizer application. Smoothed peak magnitudes and dates should therefore be interpreted as descriptive features of the processed series.

In the five-point moving-average series, leaf-extract ammonium concentration reached 32.87 mM on 26 February, compared with 16.51 mM on 25 March, approximately 50% of the February maximum (Figure 1). Leaf-extract pH was 6.03 on 26 February and 5.61 on 2 March, while the corresponding unsmoothed ammonium concentrations were 40.21 and 33.97 mM. These values yielded unsmoothed Γ_extract_ values of 43,087 and 13,837, respectively. Thus, the unsmoothed ammonium and Γ_extract_ maxima both occurred on 26 February. The later maximum in the smoothed Γ_extract_ series reflects the separate moving averaging of the ammonium and Γ_extract_ observations and should not be interpreted as a delayed physiological response.

Excluding the initial sample, which had a distinctly lower pH of 4.05, leaf-extract pH ranged from 4.61 to 6.30, with a mean of 5.31 during the subsequent sampling period.

LAI was 0.97 on 18 February and 1.10 on 26 February, close to the first fertilizer application, compared with 2.14 on 23 March, the date of the second application (Figure 1). The latter value was approximately 1.95 times the former. LAI subsequently reached 3.66 on 20 April and varied between 2.66 and 3.29 during the May measurements preceding the end of leaf sampling. Between 11 and 21 May, Γ_extract_ was approximately 355–396, and ammonium concentration was 4.48–4.77 mM. Thus, the early-season peak in the chemical indicators coincided with a substantially smaller canopy.

The measurement dataset comprises 28 dated Γ_extract_ and ammonium entries and 16 LAI measurement dates. Only date-level LAI means were available, so variability among the seven locations could not be calculated. Smoothed chemical entries and successive sampling dates were not treated as independent treatment replicates, and no significance test was applied to the contrast between the two fertilizer-associated maxima.

### 2.2. Semi-Quantitative Estimates of Net NH_3_ Exchange

**Figure 2:**
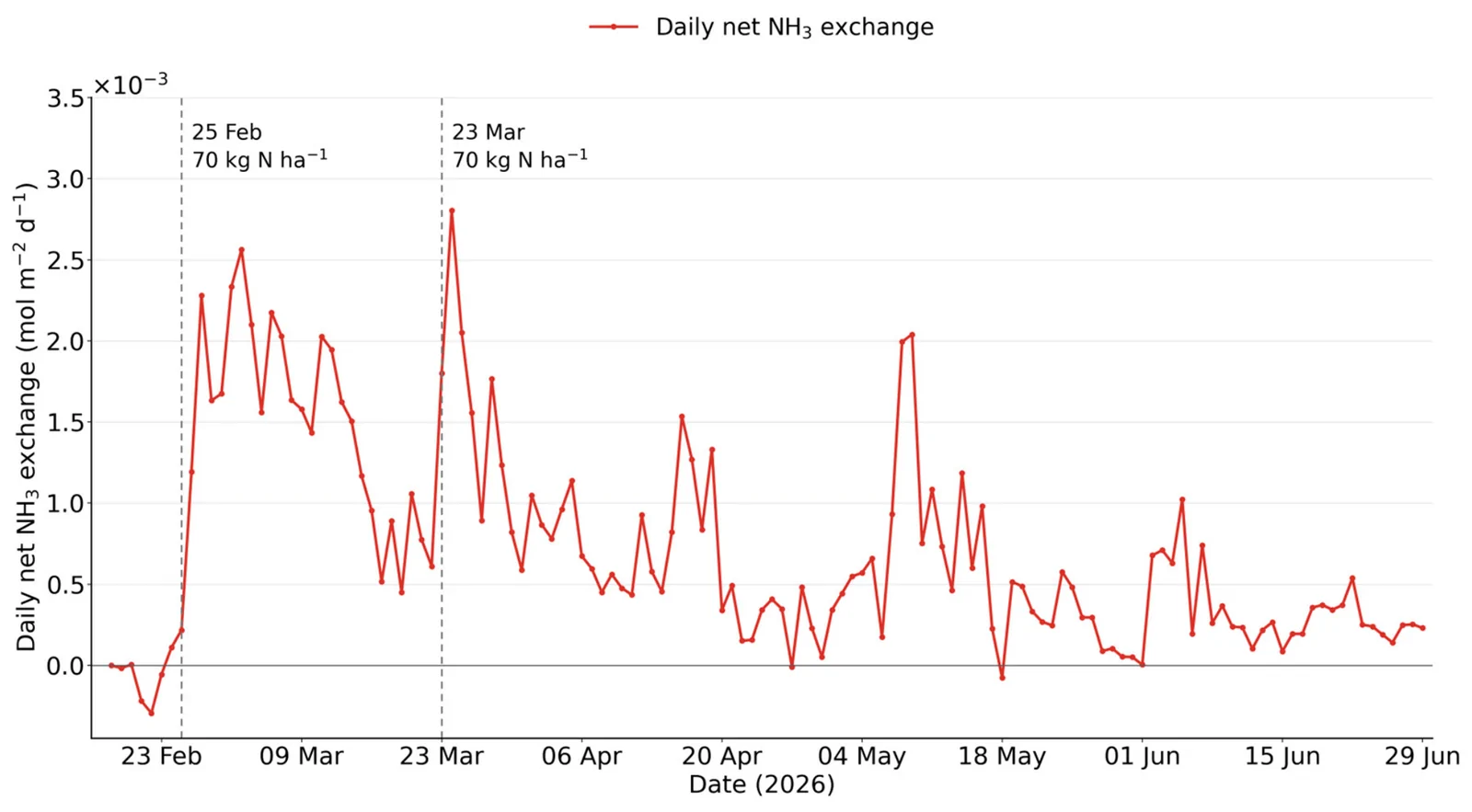
Semi-quantitative estimates of daily net NH_3_ exchange over winter oilseed rape from 18 February to 29 June 2026. Daily totals were calculated by integrating two-minute concentration gradients multiplied by the negative of the corresponding half-hourly turbulent diffusion coefficient (K_c_), using a repeating mean diurnal K_c_ profile determined at the same site during August–October 2025. Positive values indicate net emission and negative values net deposition, representing the combined exchange of soil and vegetation. Estimated cumulative net NH_3_ emission over the measurement period was 13.6 kg N ha^−1^. The use of a diffusion coefficient profile from a different season and year introduces uncertainty into the daily magnitudes and cumulative total. Daily exchange totals are expressed in mol NH_3_ m^−2^ d^−1^, whereas cumulative net emission is expressed as kg N ha^−1^.

The semi-quantitative daily net NH_3_ exchange series showed increased emission following both UAN applications (Figure 2). Sustained elevated emission followed the February application, while a renewed increase occurred after the March application. Further emission episodes occurred in April and May, including periods when Γ_extract_ was already low. Estimated cumulative net emission from 18 February to 29 June 2026 was 13.6 kg N ha^−1^. Positive daily totals represent net emission, and negative totals indicate net deposition. These values describe the combined exchange of soil and vegetation and are not estimates of the stomatal component or fertilizer-derived nitrogen loss.

### 2.3. Context from Published Emission Potentials

The descriptive summary of selected literature values from Zhang et al. [6] gave a median apoplastic Γ_s_ of 735 for fertilized vegetation and 290 for forests and semi-natural vegetation (Table 1). Arithmetic means exceeded medians in both groups, reflecting strongly right-skewed distributions. Representative high values in the fertilized group reached 40,000. The early-season Γ_extract_ maximum of approximately 24,409 is within the numerical range represented in this compilation, although correspondence in scale does not demonstrate that the same leaf compartment was sampled.

The compilation is unweighted and includes heterogeneous measured and model-derived entries. The explicitly unfertilized grassland value of 585 was assigned to the comparison group. Each separately listed value was treated as an individual entry. Reported central estimates were used when available; otherwise, the midpoint of the reported range was used. For the widest range, extending from just above zero to approximately 40,000, a representative value of 20,000 was used.

One-sided bounds, the ambiguous <400–approximately 1600 entry, and PPT-treated weeds were excluded. Quartiles were obtained by linear interpolation. Entry counts are not numbers of independent experiments. The comparison group includes observations under elevated nitrogen loading or experimental nitrogen treatments and is therefore not uniformly unfertilized. These summaries are contextual and are not an experimental estimate of fertilization effects.

## 3. Discussion

### 3.1. Canopy Development and Contrasting Responses to Equal Nitrogen Inputs

The two equal nitrogen applications were followed by different maxima in both Γ_extract_ and ammonium concentration. The first application occurred when LAI was approximately 1.10, whereas LAI was 2.14 at the second application. These observations support interpreting the larger early-season chemical response in relation to the smaller canopy, rather than nitrogen dose per unit ground area alone.

For context, dividing the applied dose of 7 g N m^−2^ ground area by the available LAI gives approximately 6.38 g N m^−2^ leaf area near the first application and 3.27 g N m^−2^ leaf area at the second. The first estimate uses LAI measured on 26 February, one day after application. This comparison describes applied nitrogen relative to estimated canopy leaf area; it does not measure nitrogen absorbed by leaves. The higher early-season ammonium concentration and Γ_extract_ suggest a temporary excess of nitrogen supply relative to canopy size and assimilation demand [7,10,11]. LAI provides evidence of canopy development, but biomass, nitrogen uptake, and assimilation rates were not measured, so the proposed mechanism remains an interpretation of the seasonal association.

The chemical contrast is also compatible with effects of temperature, radiation, and water availability on nitrogen uptake and metabolism. Because the applications occurred at different times in a single field, canopy development cannot be separated experimentally from these seasonal factors.

The spring observations followed an autumn nitrogen application of 30 kg N ha^−1^ [16]. Consequently, nitrogen supply during spring may have included residual fertilizer nitrogen and nitrogen retained in soil or plant pools during autumn, in addition to the spring applications. The preceding autumn exchange measurements provide useful management context but do not quantify the mineral nitrogen available at the beginning of the 2026 observation period. The contributions of these nitrogen pools therefore cannot be separated from the responses associated with spring fertilization.

In a broader agricultural context, the observed leaf-extract chemistry should be considered alongside soil nitrogen availability, crop nitrogen uptake, and environmental constraints. Soil fertility influences nitrogen supply, while temperature and water availability may modify the balance between uptake, assimilation, and canopy development. However, neither Γ_extract_ nor net NH_3_ exchange alone provides a measure of nitrogen use efficiency (NUE). Evaluating NUE requires crop nitrogen uptake or yield relative to a clearly defined nitrogen input or supply. The present observations therefore motivate future studies combining soil fertility indicators, crop nitrogen recovery, yield, and abiotic stress measurements to assess whether the seasonal chemical and exchange patterns are associated with differences in nitrogen utilization.

Studies in calcareous soils illustrate how nutrient availability and fertilizer effectiveness depend on soil conditions and amendment practices. Mihoub et al. [17] examined improved phosphorus nutrition in wheat, while Mihoub et al. [18] investigated the persistence of available phosphorus following modified-biochar application.

These findings provide a broader nutrient-management context, although they do not directly establish mechanisms of nitrogen recovery, NH_3_ exchange, or abiotic stress mitigation in the present study.

The maxima describe the five-point moving-average series. With irregular sampling, a five-observation window does not correspond to five days, and smoothing can alter peak magnitude and apparent timing. The observed dates therefore should not be interpreted as exact physiological response delays.

The higher chemical emission potential in the smaller early-season canopy raises the possibility of greater ammonia loss per unit leaf area under otherwise comparable exchange conditions [8]. Canopy-scale loss also depends on total leaf area, temperature, conductance, atmospheric ammonia, and other exchange pathways, so a higher Γ_extract_ does not establish a larger field-scale loss. A practical hypothesis is that smaller or more finely split early-season doses, matched to crop nitrogen demand, could limit transient ammonium accumulation and emission potential. The present study did not compare application strategies and therefore cannot identify an optimum schedule or quantify mitigation.

Recent studies provide complementary evidence linking plant nitrogen status, fertilizer management, and NH_3_ losses. In maize, Li et al. [19] found that leaf NH_3_ emission rates were associated with apoplastic NH_4_^+^ concentration and physiological traits and increased following nitrogen application. Zhang et al. [20] reported greater soil and canopy NH_3_ emissions under a single nitrogen application than under split applications, with canopy emissions related to leaf area and apoplastic NH_4_^+^ concentration. In an oilseed rape–maize rotation, Yao et al. [21] showed that fertilizer formulation and straw mulching influenced NH_3_ volatilization, while differences in nitrogen use efficiency among fertilizer treatments were not significant during the oilseed rape season. Together, these findings support considering leaf chemistry, canopy development, and soil processes jointly when interpreting NH_3_ exchange, while evaluating emission reduction and crop nitrogen recovery as distinct outcomes.

The field-scale exchange estimates provide complementary evidence of increased net emission following both applications (Figure 2). However, the contrast between the two Γ_extract_ maxima cannot be interpreted as a corresponding ratio of field-scale emissions.

According to Equation (1), the NH_3_ compensation point concentration depends on temperature as well as on the NH_4_^+^/H^+^ ratio: at a given ratio, increasing temperature raises the equilibrium gas-phase NH_3_ concentration. Warmer conditions following the March application could therefore partly offset the lower Γ_extract_ and help explain why the highest estimated daily net emission occurred after the second application.

Soil volatilization, canopy uptake and release, and atmospheric transport all contribute to net exchange. The additional emission episodes in May, when Γ_extract_ was low, further indicate that the leaf-extract ratio alone does not describe the temporal variation in net field-scale exchange.

### 3.2. Chemical Meaning of the Emission Potential

The pronounced seasonal variation in Γ_extract_ indicates that a single fixed value would not capture the temporal pattern observed in this study. However, Γ_extract_ is a chemical indicator rather than a flux measurement; estimating stomatal ammonia exchange from this indicator additionally requires leaf temperature, ambient ammonia concentration, and stomatal conductance [8]. The higher extract pH values relative to the initial sample indicate lower hydrogen ion activity, which would increase Γ_extract_ at a given ammonium concentration. However, this seasonal pH difference has no confirmed causal link to fertilization. The flux estimates in Figure 2 were derived independently of Γ_extract_ from measured atmospheric concentration gradients and an assumed turbulent diffusion coefficient profile. Their inclusion does not validate Γ_extract_ as an in vivo stomatal emission potential or establish a quantitative Γ_extract_–flux relationship.

### 3.3. Comparison with Other Vegetation Studies

Published emission potentials vary substantially among vegetation types and nitrogen regimes [6,12]. The contextual medians in Table 1 are much lower than the early-season maximum in this study, whereas the upper representative values extend to a comparable magnitude. This highlights the distinction between transient peaks and central or background values. A high value following fertilization should not be used as a characteristic parameter for the entire crop season.

More recent studies have examined ammonia exchange in a pine forest and an Amazonian rainforest [22,23]. These studies extend the environmental contexts available for comparison, but forest exchange cannot serve as a direct reference for the response of a fertilized arable crop. Differences in canopy structure and nitrogen supply and exchange pathways must be retained when interpreting apparent similarities or differences. Seasonal observations in oilseed rape address a more specific question: how an extracted leaf–fluid ratio evolves across repeated spring nitrogen inputs.

The low late-season Γ_extract_ values overlap in scale with several central literature estimates. Their occurrence alongside lower ammonium concentrations and a larger canopy provides seasonal context, but numerical plausibility alone does not validate the apoplastic specificity of the extracts.

### 3.4. Extraction Specificity and Limits of Interpretation

The recovery procedure was adapted from published leaf–fluid extraction methods [9,10,13], but the use of frozen tissue without infiltration represents an important departure from validated apoplastic sampling procedures.

The physical limitation of the present extraction procedure is that fluid recovered from frozen and thawed leaves cannot be assumed to originate exclusively from the apoplast. Freezing and thawing may compromise membrane integrity, allowing intracellular ammonium and buffering compounds to enter the recovered fluid. Larger leaf pieces reduce cut-edge exposure but do not prevent freezing-related loss of compartment separation. In contrast, Husted and Schjoerring [9] developed a vacuum-infiltration procedure for recovering apoplastic solution, quantified dilution using an extracellular tracer, and assessed cytoplasmic contamination using malate dehydrogenase activity. Their validation cannot be transferred directly to the present procedure, which used frozen tissue without infiltration or an independent contamination assay.

The mathematical limitation is that Γ_extract_ = [NH_4_^+^]_extract_/[H^+^]_extract_ is the ratio in the recovered mixture, not necessarily the ratio in the native apoplast. Even under a simple volume-weighted mixing assumption for ammonium, the apoplastic concentration cannot be recovered without knowing the contributions and compositions of the different compartments. Moreover, pH is logarithmic, and the pH of mixed fluids depends on acid–base equilibria and buffering capacities rather than the arithmetic mean of their original pH values. Consequently, Γ_extract_ is not a volume-weighted average of compartment-specific emission potentials. At a fixed ammonium concentration, an increase of one pH unit increases the calculated Γ_extract_ tenfold. Intracellular contributions can therefore alter both the numerator and denominator, and neither the magnitude nor the direction of the resulting bias can be established from the present measurements. Γ_extract_ is accordingly interpreted as an operational leaf-extract indicator, without assuming equivalence to apoplastic Γ_s_ or using it directly to calculate a stomatal compensation point.

The absence of infiltration solution avoids added-solvent dilution but likewise does not prove apoplastic specificity.

Apoplastic purity was not independently assessed in the present extracts. A subsequent comparison with fresh-leaf apoplastic washing fluid, supported by appropriate contamination controls, would help determine the relationship between Γ_extract_ and Γ_s_. Malate dehydrogenase activity can be useful in such assessments, but some species have endogenous apoplastic activity, so interpretation requires a relevant baseline and intracellular reference rather than a simple presence-or-absence test [13]. Tests on stored extracts additionally need controls for preservation of enzyme activity during storage.

Analytical and biological sources of uncertainty should also be separated. Small-volume pH determination requires adequate calibration and temperature control, and pH represents hydrogen ion activity rather than concentration exactly [14]. Measurements made directly in undiluted extracts avoid the need for a dilution-based back-calculation; the latter is a separate methodological approach [15]. Nevertheless, the measured extract pH should not be assumed to be identical to native apoplastic pH after freezing, thawing, and recovery.

The results establish seasonal associations in the extracted-fluid indicator and provide semi-quantitative estimates of net field-scale NH_3_ exchange. They do not establish a causal fertilization effect, isolate stomatal flux, or provide a fertilizer-specific emission factor. Because the three extracts collected on each sampling date were pooled before analysis, within-date variability in leaf chemistry could not be quantified. Preserving this distinction is necessary for any later statistical analysis.

The joint LAI, leaf chemistry, and field-scale exchange record provides a basis for testing whether fertilizer inputs matched more closely to canopy development and crop nitrogen demand would reduce transient increases in emission potential and ammonia loss. Paired fresh- and frozen-leaf sampling, independent validation of extract purity, and flux calculations based on contemporaneous turbulence measurements would allow the proposed physiological and management implications to be evaluated.

The exchange estimates have an additional limitation: they use a repeating mean diurnal Kc profile obtained during August–October 2025 rather than turbulence measurements from February–June 2026. Differences in meteorology, atmospheric stability, canopy structure, and aerodynamic conditions may affect the applicability of this profile. A repeating profile cannot reproduce day-to-day changes in turbulent transport, so apparent daily emission responses may partly reflect meteorological variability that is not captured in the calculation. Consequently, both daily exchange totals and the cumulative estimate of 13.6 kg N ha^−1^ should be treated as semi-quantitative. The lack of an unfertilized control also prevents separation of background exchange from fertilizer-associated loss.

## 4. Materials and Methods

### 4.1. Field Site and Crop Management

The study was conducted in an arable plot near Kartal, Hungary, at the Gödöllő Experimental Farm (47°39′42.56″ N, 19°31′43.21″ E; approximately 153 m above sea level). The regional climate is continental, with a reported long-term mean annual precipitation of 550–600 mm for 1991–2020. The broader field observation period extended from 18 February to 30 June 2026, during which reported cumulative precipitation was 115 mm. This meteorological period is longer than the leaf-sampling period and should not be interpreted as the duration of the leaf-extract record.

The soil was classified as a Haplic Phaeozem (Humic, Pantoloamic). Particle-size fractions were 32.3% clay, 49.1% silt, and 18.6% sand, corresponding to a silty clay loam texture in the USDA system. Winter oilseed rape (*Brassica napus* L.) was sown on 26 August 2025.

Before crop establishment, the field received 200 kg ha^−1^ of granular diammonium phosphate on 25 August 2025, corresponding to a reported nitrogen input of 30 kg N ha^−1^ [16]. Net NH_3_ exchange following this application was monitored at the same site from 28 August to 21 October 2025 [16].

Terrasol (urea–ammonium nitrate, UAN) liquid fertilizer containing 24% N and 6% SO_3_ was applied on 25 February and 23 March 2026 at a product rate of 292 kg ha^−1^ on each occasion, supplying 70.08 kg N ha^−1^ per application. The fertilizer was applied undiluted in February and diluted 1:1 (*v*/*v*) with water in March. Both applications were made using a high-clearance sprayer equipped with nozzles designed for liquid fertilizer application. Dilution water was additional to the fertilizer product rate. The total spring nitrogen input was 140.16 kg N ha^−1^.

### 4.2. Leaf Sampling and Fluid Recovery

Leaves were collected during the late morning between 18 February and 21 May 2026. Sampling covered different field locations and canopy heights to represent variation in leaf development and shading. Leaves were not restricted to a single canopy position or age class. On each sampling occasion, approximately three sets of 6–13 g of leaf material were collected and stored frozen at −18 °C until processing. The frozen leaf samples were thawed at 4 °C over several hours before centrifugation. Leaf pieces were placed loosely in the centrifuge tubes, avoiding compaction and mechanical damage. The three leaf samples collected on each sampling occasion were centrifuged separately, and the resulting extracts were pooled before analysis. In total, 28 pooled extracts were analyzed, each corresponding to one sampling date. The unsmoothed ammonium concentrations, pH, and Γ_extract_ values are provided in Supplementary Dataset S1. Because the three extracts obtained on each sampling date were pooled before chemical analysis, within-date variability in ammonium concentration, pH, and Γ_extract_ could not be quantified.

The recovery procedure was adapted from Husted and Schjoerring [9] and Mattsson and Schjoerring [10], with reference to the centrifugation-based approach described by O’Leary et al. [13]. Larger leaf pieces were used instead of small segments to reduce the contribution of cut-edge damage. No infiltration or isotonic or buffer solution was added. The frozen leaf samples were thawed at 4 °C over several hours before centrifugation. Leaf pieces were placed loosely in the centrifuge tubes, avoiding compaction and mechanical damage. Leaf material was centrifuged for 5 min at 3300 rpm (1534× *g*) and 5 °C using a Thermo Fisher Scientific centrifuge equipped with a HIGHConic II fixed-angle rotor. The mean mass of leaf material processed was 2.99 g, and the mean mass of recovered fluid was 0.77 g. The ratio of these mean masses corresponds to approximately 0.258 g of recovered fluid per gram of leaf material.

These centrifugation conditions were selected to limit additional mechanical damage during fluid recovery.

Previous work provides evidence that apoplastic fluid can be recovered with low cytoplasmic contamination under validated extraction conditions. In oilseed rape, Husted and Schjoerring [9] reported malate dehydrogenase (MDH) activity in the recovered apoplastic solution below 1% of that in bulk leaf extracts, supporting low contamination during their vacuum-infiltration procedure. O’Leary et al. [13] likewise describe MDH activity as an indicator of intracellular contamination, while noting that endogenous apoplastic activity may require a species-specific baseline. These findings support the methodological basis of centrifugation-based recovery but do not establish the purity of the present extracts because leaves were stored frozen and recovered without infiltration. No MDH assay was performed in this study; therefore, the recovered fluid is described as leaf extract, and possible intracellular contributions cannot be excluded.

### 4.3. Ammonium and pH Measurements

The pH of undiluted leaf extracts was measured with a Metrohm 6.0234.110 combined micro pH electrode.

Ammonium concentration was determined by ion chromatography. Before analysis, the samples were filtered through a 0.22 µm PVDF-L syringe filter (FilterBio, Nantong, China). The samples were acidified to pH < 5 with H_2_SO_4_ solution to minimize ammonia volatilization and diluted with Milli-Q water. The concentration of NH_4_^+^ was measured by ion chromatography using a Prominence LC-20AD system (Shimadzu, Kyoto, Japan) equipped with a Shodex IC YS-50 column (4.6 × 125 mm), maintained at 40 °C, and a conductivity detector (CDD-10A VP). The eluent was 4.0 mmol L^−1^ methanesulfonic acid. The flow rate was maintained at 0.3 mL min^−1^ for the first 11 min and then increased to 1.0 mL min^−1^. The injection volume was 20 µL, and the total run time was 16 min. The retention time of NH_4_^+^ was 9.5 min. Ammonium concentrations were determined using a calibration curve, and the limit of quantification (LOQ) was 0.01 mM.

The ammonium and pH measurements used to calculate Γ_extract_ were obtained from the same extract.

### 4.4. Definition of Compensation Point Concentration

The stomatal compensation point concentration of NH_3_ can be expressed using a formula combining Henry’s law and ammonium dissociation equilibria [24,25]:

(1) $$\left[{NH}_{3}\right]=K_{H}\times K_{A}\times \left\{exp\left[\frac{{∆H}_{H}^{0}+{∆H}_{A}^{0}}{R}\left(\frac{1}{298.15}-\frac{1}{T}\right)\right]\right\}\times \Gamma ,$$

where K_H_ and K_A_ are the Henry’s law constant for NH_3_ partitioning between apoplastic fluid and the atmosphere and the ammonium dissociation constant, respectively, and ${∆H}_{H}^{0}$ and ${∆H}_{A}^{0}$ (J mol^–1^) are the corresponding standard enthalpy changes, *T* is the absolute temperature (K), and R is the universal gas constant (8.314 J mol^−1^ K^−1^). The dimensionless emission potential, Γ = [NH_4_^+^]/[H^+^], determines the stomatal compensation point at a given temperature. In this study, the ratio calculated from leaf extracts is denoted Γ_extract_.

### 4.5. Leaf Area Index Measurements

LAI was measured on 16 dates from 18 February to 8 June 2026 with an AccuPAR LP-80 PAR/LAI ceptometer (METER Group, Pullman, WA, USA) at seven field locations per date. For each measurement date, LAI was averaged across the seven field locations. LAI was used to describe canopy development and contextualize the leaf-extract chemistry; it was not converted into biomass or interpreted as a direct measurement of nitrogen demand.

### 4.6. Temporal Processing and Descriptive Comparison

For each pooled leaf extract, hydrogen ion concentration was approximated from the directly measured pH as [H^+^] = 10^−pH^ mol L^−1^, assuming that hydrogen ion activity could be approximated by concentration. Γ_extract_ was then calculated as [NH_4_^+^]/[H^+^], with both concentrations expressed in mol L^−1^. Each Γ_extract_ value was calculated from the ammonium concentration and pH of the same pooled extract before temporal smoothing. The original, unsmoothed Γ_extract_ values are presented alongside the five-point moving-average series in Figure 1.

The Γ_extract_ and ammonium time series were then smoothed using a centered five-point moving average. At the beginning and end of each series, where a complete five-point window was unavailable, the window was reduced to four and then three observations toward the endpoints. The resulting series were plotted without further smoothing or adjustment.

Leaf chemistry and LAI were analyzed as separate time series because their measurement dates did not always coincide. LAI was retained as the mean of the seven locations, without smoothing. Lines connect available dates as visual guides; no chemical values were extrapolated beyond 21 May. Maxima and ratios were calculated from the dated values. Nitrogen input relative to estimated leaf area was calculated as applied N per unit ground area divided by LAI; this is a descriptive loading index, not a measure of leaf nitrogen uptake. No inferential tests, confidence intervals, or decay fits were calculated from the smoothed values.

### 4.7. Flux Measurements

Ammonia flux measurements were performed using the aerodynamic gradient method [16]. Ammonia mixing ratios were measured at two heights, above the canopy surface, using a quantum-cascade-laser photoacoustic analyzer operating at 10.39 µm. Air was sampled alternately from the two levels through identical 4 m PTFE sampling lines at a flow rate of 800 cm^3^ min^−1^. At each level, a 1 min flushing period was followed by 1 min of data acquisition. Identical tubing materials and lengths, together with flushing before data acquisition, were used to minimize sampling-related differences and adsorption–desorption effects. The photoacoustic cell was maintained at 50 °C, and the minimum detectable NH_3_ mixing ratio was approximately 3 ppb with 2 min averaging. Mixing ratios were converted to concentrations (nmol m^−3^), from which vertical concentration gradients were calculated.

Fluxes were subsequently estimated from the concentration-gradient series measured between 18 February and 29 June 2026 using the mean diurnal profile of the turbulent diffusion coefficient determined at the same site during August–October 2025. The resulting daily net NH_3_ exchange totals are presented in Figure 2. Because turbulent diffusion coefficients specific to the 2026 measurement period were not yet available, these flux estimates should be regarded as semi-quantitative. Nevertheless, they provide a basis for assessing the timing and approximate magnitude of emission responses associated with fertilizer application. These estimates were calculated independently of Γ_extract_. They represent net exchange over the soil–vegetation system and cannot partition soil, stomatal, and non-stomatal pathways. The cumulative net exchange is therefore not interpreted as a fertilizer-specific emission factor.

### 4.8. Use of Generative Artificial Intelligence

During preparation of this manuscript, the authors used ChatGPT (GPT-5.6; OpenAI) to assist with drafting, English language editing, organization of the text, and preparation of the figure from the measured data. The authors reviewed and edited the text, verified the figure against the underlying data, and take full responsibility for the content of the publication.

## 5. Conclusions

Winter oilseed rape showed a stronger early-season leaf-extract chemical response following the first of two equal spring nitrogen applications. The five-point moving-average Γ_extract_ series reached approximately 24,409 after the February application, compared with 8533 after the March application. Moving averages of ammonium maxima were 32.87 and 16.51 mM, respectively. LAI near the first application was 1.10, compared with 2.14 at the second, supporting the interpretation that the same field-scale dose represented a greater nitrogen input relative to canopy leaf area early in the season.

The observations suggest a temporary imbalance between nitrogen supply and crop development and motivate testing smaller or more finely split early-season applications matched to crop nitrogen demand. The semi-quantitative flux series showed increased net NH_3_ emission following both nitrogen applications, with an estimated cumulative net emission of 13.6 kg N ha^−1^ from 18 February to 29 June 2026. This estimate depends on the use of a mean diurnal turbulent diffusion coefficient profile from August–October 2025 and represents combined soil–vegetation exchange. It neither isolates stomatal emission nor establishes fertilizer-derived loss. Additional emission episodes during periods of low Γ_extract_ show that the leaf-extract indicator alone cannot explain net field-scale exchange. Because the fluid was recovered from frozen leaves without independent apoplastic validation, Γ_extract_ remains a leaf-extract indicator. The absence of an unfertilized control and replicated treatments precludes causal attribution and conclusions about mitigation efficacy or an optimum nitrogen dose. Contemporaneous turbulence data, validated apoplastic sampling, and replicated management comparisons are needed to test these interpretations.

## Figures and Tables

**Table 1:** Descriptive compilation of representative Γ_s_ values from Zhang et al. [6]. All emission potentials are dimensionless.

Statistic	Forests and Semi-Natural Vegetation	Fertilized Vegetation
Number of representative entries	32	58
Minimum	0.8	38
Maximum	8500	40,000
Arithmetic mean	1437	3374
Median	290	735
Sample standard deviation	2289	7683
First quartile	146.6	353.4
Third quartile	1465	2572
Geometric mean	339.4	890.9

## Data Availability

The data presented in this study are available in Supplementary Dataset S1.

## Supplementary Materials

The following supporting information can be downloaded at: https://www.mdpi.com/article/10.3390/plants15213411/s1, Dataset S1: Leaf-extract ammonium, pH, emission potential, and leaf area index data.
